# Long term OSLD reader stability in the ACDS level one audit

**DOI:** 10.1007/s13246-014-0320-7

**Published:** 2014-12-14

**Authors:** Andrew D. C. Alves, Jessica Lye, John Kenny, Leon Dunn, Joerg Lehmann, Andrew Cole, Tomas Kron, Duncan Butler, Peter Johnston, Ivan Williams

**Affiliations:** 1 Australian Clinical Dosimetry Service, Yallambie, VIC 3085 Australia; 2 Peter MacCallum Cancer Centre, Melbourne, 3008 Australia; 3 Australian Radiation Protection and Nuclear Safety Agency, Yallambie, VIC 3085 Australia

**Keywords:** Radiotherapy, Optically stimulated luminescence dosimeter (OSLD), OSLD readout uncertainty, OSLD element correction factor, Level one dosimetric audit

## Abstract

The Australian Clinical Dosimetry Service (ACDS) has demonstrated the capacity to perform a basic dosimetry audit on all radiotherapy clinics across Australia. During the ACDS’s three and a half year trial the majority of the audits were performed using optically stimulated luminescence dosimeters (OSLD) mailed to facilities for exposure to a reference dose, and then returned to the ACDS for analysis. This technical note investigates the stability of the readout process under the large workload of the national dosimetry audit. The OSLD readout uncertainty contributes to the uncertainty of several terms of the dose calculation equation and is a major source of uncertainty in the audit. The standard deviation of four OSLD readouts was initially established at 0.6 %. Measurements over 13 audit batches—each batch containing 200−400 OSLDs—showed variability (0.5−0.9 %) in the readout standard deviation. These shifts have not yet necessitated a change to the audit scoring levels. However, a standard deviation in OSLD readouts greater than 0.9 % will change the audit scoring levels. We identified mechanical wear on the OSLD readout adapter as a cause of variability in readout uncertainty, however, we cannot rule out other causes. Additionally we observed large fluctuations in the distribution of element correction factors (ECF) for OSLD batches. We conclude that the variability in the width of the ECF distribution from one batch to another is not caused by variability in readout uncertainty, but rather by variations in the OSLD stock.

## Introduction

Performing an independent and standardised audit is an internationally recognised way to minimise the risk of a dosimetric error in radiotherapy practice [1]. The Australian Clinical Dosimetry Service (ACDS) conducts a level one postal audit using optically stimulated luminescence dosimeters (OSLD, nanoDots (Landauer, Inc., Glenwood, IL), encased in Perspex blocks to determine the absorbed dose to water per monitor unit for MV photon and electron beams under reference conditions. The audit is based on the well-established methodology of imaging and radiation oncology core (IROC) Houston QA Center (formerly Radiological Physics Center (RPC)) [2] and is explained in detail by Lye et al. [3]. The characterisation of OSLDs for use in clinical dosimetric measurements has been reported by Jursinic [4], by the International Atomic Energy Agency [5], and undertaken specifically in the context of the ACDS audit by Dunn et al. [6].

A key component of any audit is the pass/fail tolerance. The ACDS level one audit tolerance was defined using a rigorous uncertainty calculation [3]. It is important for the ACDS to monitor the individual uncertainty components and ensure that they do not drift over time. A significant uncertainty component that could be susceptible to drift is the OSLD MicroStar (Landauer, Inc., Glenwood, IL) reader stability. In this technical note we monitor multiple OSLD read outs, specifically the standard deviation of four reads, over a period of 3 years since mid-2011. We examine a large data set from field trial audit batches and fully commissioned audit batches. The field trial phase was used to establish the audit methodology and uncertainty which was then followed by the fully commissioned phase where audit scores were generated from the audit results and known uncertainty. The inclusion of electron beams in the most recent batches has increased the number of OSLDs required to perform an audit. We also anticipate a growth in the number of Australian radiotherapy linacs, and there is potential to use OSLDs to perform higher lever audits or quality assurance beyond basic dosimetry [7, 8] similar to the use of radiochromic film and thermoluminescent dosimetry capsules in higher level audits [9]. It is timely to establish protocols to examine an OSLD reader’s long term stability, under an ever increasing workload.

## Method

The ACDS performed quarterly OSLD mail outs to an ensemble of facilities. For each quarterly mail out a batch of OSLDs was prepared. The readout analysis was performed independently for each batch. Field trails and early audit batches contained 200−400 OSLDs. Since the inclusion of electron audits in September 2013 all batches have contained 400−450 OSLDs.

An initial read on each OSLD determined the signal from the un-irradiated OSLD, *read*_*(un-irr)*_. The batch was then exposed to 1 Gy using an Eldorado Co-60 unit. To ensure uniform dose to all OSLDs, groups of eight OSLDs were located in a 2.5 cm radius ring around the centre of a 10 × 10 cm^2^ field at 5 cm depth in solid water (20 cm backscatter) with a source to surface distance (SSD) of 100 cm. The raw OSLD signal, proportional to the emitted light of the OSLD, *read*_*j*_, was corrected for reader depletion of 0.03 % per read [6]. An average of the signal from four reads was taken and the initial un-irradiated read value was subtracted to return the value *counts*_*(bg)*_ (Eq. 1). The letter *j* in Eq. 1 represents the *j*th readout on a single OSLD.

1 $$counts_{(bg)} = \frac{{\sum\limits_{j = 1}^{4} {\frac{{read_{j} }}{{e^{{ - 3 \times 10^{ - 4} }} j}}} }}{4} - read_{(un - irr)}$$

The normalised standard deviation of the four reads, *σ*_*read*_, and element correction factor, *ECF*, (Eq. 2) for each OSLD was determined. The letter *i* in Eq. 2 represents the *i*th OSLD in a batch of *n* OSLDs.

2 $$ECF_{i} = \frac{{\sum\limits_{i = 1}^{n} {counts_{(bg)i} } }}{{n \times counts_{(bg)i} }}$$

In the field trial (batches 4−7) the set of four reads was performed once. To decrease the audit uncertainty in subsequent batches (batches 8−16), the read outs were performed in two separate sets to make a total of eight reads. This paper intends to find if the mean *σ*_*read*_ value in a batch has remained constant since the conclusion of the field trials and to also gauge how shifts in mean *σ*_*read*_ will affect the audit’s uncertainty budget.

The audit results are determined by the deviation of the facility stated dose from the ACDS measured dose, defined in Eq. 3 where *D*_*fac*_ is the dose quoted from the facility under audit and *D*_*ref*_ is the dose determined by the ACDS found using Eqs. 4 and 5:

3 $${\text{Audit Result}}\,{ = }\frac{{D_{fac} \, - D_{ref} }}{{D_{ref} }}$$

4 $$D_{ref} = D_{audit} \cdot BF$$

5 $$D_{audit} = \left[ {\left( {Counts \times k_{r} - Counts_{{\left( {bg} \right)}} \times k_{{r\left( {bg} \right)}} \times k_{{f\left( {bg} \right)}} } \right) \times k_{f} } \right] \times ECF \times S \times k_{E} \times k_{L}$$

The block factor (BF), converts *D*_*audit*_, the Perspex block dose, to *D*_*ref*_, the dose in water under reference conditions. *Counts* is the output of the OSLD reader after facility irradiation, averaged from eight readings and corrected for reader depletion. The subscript, *bg*, refers to a reading made on the OSLD after it is irradiated to 1 Gy in ^60^Co to determine the *ECF* prior to delivery of the audit kit to the facility. *k*_*r*_, *k*_*f*_, *k*_*E*_, and *k*_*L*_ are the reader, fading, energy, and non-linearity corrections, respectively (more detail given in Lye et al. [3]). *S* is the batch sensitivity, in cGy/counts, to 1 Gy of 6 MV photons.

The audit outcome is based on a relative combined standard uncertainty (σ_audit_) of 1.3 %. The outcome is categorised as either pass (optimal level) when the score is ≤2σ_audit_, pass (action level) when the score is >2σ_audit_, or fail when the score is >3σ_audit_. The uncertainty budget has contributions from the uncertainty in delivering dose at the facility and the uncertainty in measuring the OSLD dose at the ACDS. This budget is shown in detail in the ACDS’s previous publication [3], and in Tables 1 and 2 below. Several of the uncertainties in the measurement of the OSLD are type A [10] having been evaluated by statistical analysis through the commissioning of the audit methodology.

**Table 1:** Relative standard uncertainties for the ACDS measured dose

Quantity	ACDS relative standard uncertainty^a^	
	100 u_iA_	100 u_iB_
*Counts*	*0.22* ^b^	–
*Background counts*	*0.22* ^b^	–
Reader correction	–	0.14
Background reader correction	–	0.14
Fading correction	–	0.07
Background fading correction	–	0.07
*Element correction factor*	*0.22* ^b†^	–
*Sensitivity*	*0.50* ^b†^	–
Energy correction	0.43	–
Non-linearity correction	–	0.04
Block factor	0.44	0.30
Reference distance	–	0.20
Quadratic summation:	0.88	0.42
Combined relative standard uncertainty:	**1.0**	

^a^Sub heading definitions: 100 u_iA_ represents type A uncertainties expressed as a percentage, 100 u_iB_ represents type B uncertainties expressed as a percentage

^*b*^Uncertainties which are susceptible to drift in the reader stability

**Table 2:** Combination of uncertainties for both the ACDS measured dose and the facility measured dose. A combined relative standard uncertainty σ_audit_, associated with an ACDS Level one audit of 1.3 % is found

Quantity	ACDS relative standard uncertainty	
	100 u_iA_	100 u_iB_
ACDS measured dose	0.88	0.42
Facility measured dose	0.02	0.92
Quadratic summation	0.88	1.01
Combined relative standard uncertainty (σ_audit_)	**1.3**	

The variables *Counts*, *Counts*_*(bg)*_, *ECF*, and *S* are all determined through multiple reads of OSLDs and are susceptible to drift in the reader stability. These uncertainty components are italicised in Table 1. The uncertainties assigned to *Counts*, *Counts*_*(bg)*_, and ECF, in the *D*_*audit*_ uncertainty budget are all derived from *ECF* readout data with the assumption that the uncertainty in delivering the same dose to each OSLD in the batch is negligible. Readout uncertainty, *U*_*read*_, is defined as the normalised standard deviation of the readouts, *σ*_*read*_, divided by the square root of the number of reads.

Continued statistical analysis over many audits to evaluate the uncertainty in these variables provides a dynamic assessment of the OSLD reader’s functional stability. Wear on the reader’s mechanical parts, contamination in optical components, and faults with electronic components may all give rise to a greater uncertainty in determining the audit dose and therefore the audit outcomes must be adjusted accordingly. Further, any increase in the dose measurement uncertainty will lessen the audit’s ability to find dosimetric errors in Australian radiotherapy. It is crucial for the ACDS to be fully aware of, and take remedial action to counter, any rise in measurement uncertainty.

The batch sensitivity, *S*, is determined through multiple readouts of a sub-batch of OSLDs that have been exposed to a nominal dose of 1 Gy (6 MV photons, TPR_20,10_ = 0.673) under reference conditions (10 cm depth, 90 cm SSD) in solid water using the Australian Radiation Protection and Nuclear Safety Agency’s Elekta Synergy (Elekta AB, Stockholm, Sweden) linac. In this technical note the uncertainty in *S* is not discussed in detail, however, it is worthy to note that an increase in *U*_*read*_, will also increase the uncertainty in *S*.

## Results

**Fig. 1:**
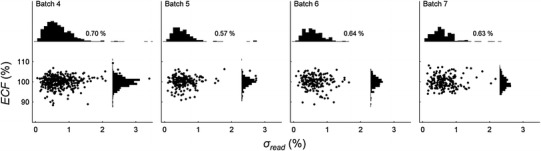
The *ECF* plotted against *σ*_*read*_ for batches 4–7 (field trial batches). The mean *σ*_*read*_ varies from 0.57 to 0.70 %. The field trial batches have established the typical range of *σ*_*read*_ that a group of four read outs produces

**Fig. 2:**
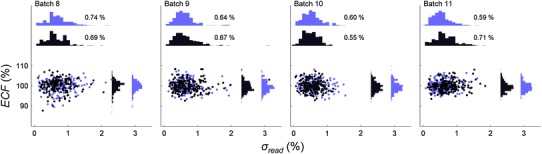
*ECF* plotted against *σ*_*read*_ for batches 8–11 (audit batches). The mean *σ*_*read*_ varies from 0.55 to 0.74 %. A similar result to the field trial batches indicates the reader is operating stably. Unlike the field trials, the ECF read was conducted on two separate sessions, shown here as two groups of data coloured *blue* and *black*

**Fig. 3:**
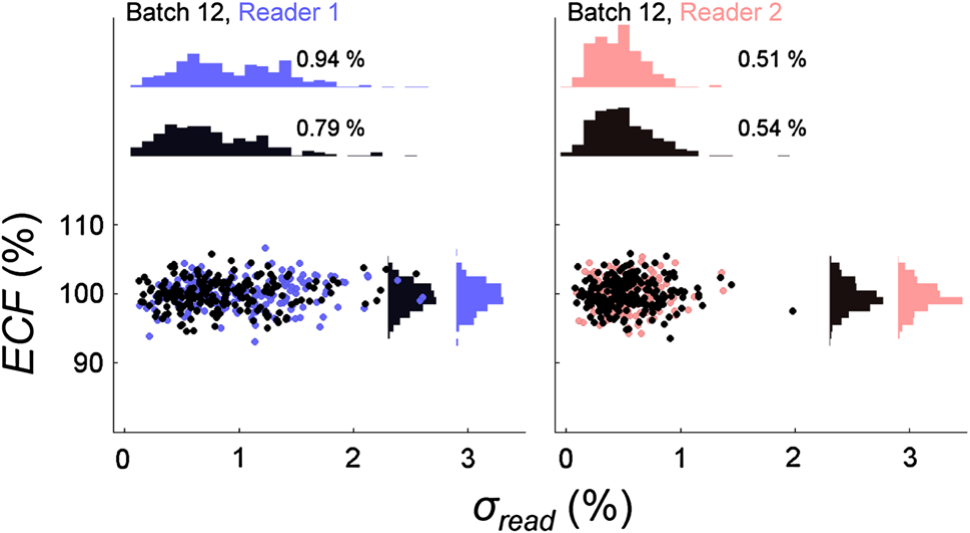
*ECF* plotted against *σ*_*read*_ for batch 12. For reader 1 the mean *σ*_*read*_ varies from 0.79 to 0.94 %. This result indicates the reader stability has deteriorated. The read outs were then performed on reader number 2 (coloured *red* and *black*) and mean *σ*_*read*_ (0.51–0.54 %) was found to be at previously accepted values

**Fig. 4:**
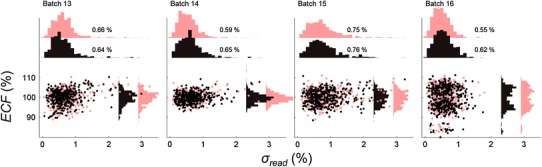
*ECF* plotted against *σ*_*read*_ for batches 13–16. The mean *σ*_*read*_ varies from 0.55 to 0.76 %. This result indicates the reader stability is maintained at previously accepted values

To visually summarise the readout results, *ECF* is plotted against *σ*_*read*_ for each OSLD in the batch in Figs. 1, 2, 3 and 4. Histograms have been inserted on the vertical and horizontal axes to show the distributions of *ECF*s and *σ*_*read*_ respectively. The inset number on the horizontal histogram is the mean of all the *σ*_*read*_ values for the batch. It was found that on occasion a single read could return an anomalous value and *σ*_*read*_ of four reads would appear as an outlier, away from the main peak. The anomalous readings are generally always low and we attribute their presence to operator error, that is, an unintentional turn of the readout dial back to home position prematurely, or a mechanical failure of the positioning dial to correctly position the OSLD in the light field.

Figure 1 displays the results for the field trial batches (4−7) showing the distributions of *σ*_*read*_. The mean *σ*_*read*_ value ranges from 0.57 to 0.70 %. The mean across all batches is 0.63 %, and the standard deviation of the *σ*_*read*_ values is 0.3 %. This number is in agreement with the reading uncertainty quoted by Mrčela et al. [11] of (0.6 ± 0.3) %. When eight reads are conducted *U*_*read*_ for the level one audit has been calculated, based on the field trail result, to be 0.22 %. The uncertainty in the mean of the *σ*_*read*_ value is ±0.02 %—very low because of the large number of OSLDs in the batches. We regard a shift in mean *σ*_*read*_ that is greater than ±3 × 0.02 % to be due to reader instability.

The first four audit batches (8−11) in Fig. 2 showed a similar stability to the field trial results with the mean *σ*_*read*_ ranging from 0.55 to 0.74 %. In batch 12, shown in Fig. 3, the mean *σ*_*read*_ was found to be 0.94 % in the second OSLD read out session. This increase is enough to change the least significant figure in the relative combined standard uncertainty for the level one audit, from 1.3 to 1.4 %. To counter this increase the second ACDS OSLD reader was examined with a new nanoDot adapter. We found that the mean *σ*_*read*_ value was reduced to 0.51−0.54 %, a level lower to that found in the audit field trials and early audits. The continued use of reader 2 in batches 13−15 showed a gradual increase in mean *σ*_*read*_ until batch 16, when a new OSLD nanoDot adapter was used.

In Fig. 6 the width of the *ECF* distribution, *ECF*_*width*_ is plotted against mean *σ*_*read*_ for all batch readouts. This width is defined as the standard deviation of all the *ECF* values in a batch. The data is plotted in this way to determine whether, or not, the distribution of *ECF* values remains constant over different batches. The *ECF*_*width*_ varies from 2 to 5 % and it is not correlated with mean *σ*_*read*_.

## Discussion

**Fig. 5:**
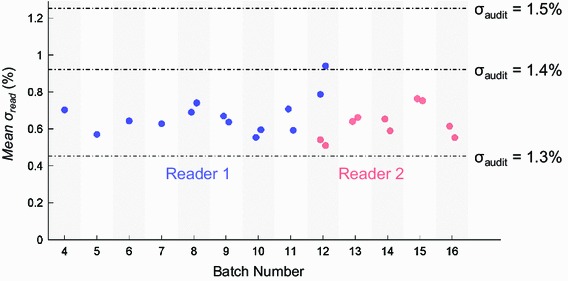
The mean *σ*_*read*_ for each readout session of each batch. The uncertainty in the mean *σ*_*read*_ value has been established and is indicated by the marker size

The reader instability is summarised in the plot shown in Fig. 5. A batch readout session returns individual *σ*_*read*_ values for each OSLD in the batch. The mean *σ*_*read*_ values have been plotted for each read out session of each batch. The mean *σ*_*read*_ values from the field trial batches was used to define *U*_*read*_ which in turn affects the relative combined standard uncertainty, σ_audit_, as shown in Tables 1 and 2. The uncertainty in the mean *σ*_*read*_ value in a single readout session has been calculated to be ±0.02 %, signified by size of the markers in Fig. 5. Because shifts in the mean *σ*_*read*_ value are greater than this uncertainty we can attribute these shifts to real variability in the process and not statistical fluctuations. Recalculated audit scoring tolerances, σ_audit_, due to an increase in *σ*_*read*_, are marked as dashed lines in Fig. 5. A mean standard deviation in OSLD readouts greater than 0.9 % will change the audit scoring levels. The result from batch 12, reader 1, are close to limit (when averaging two readout sessions), and without the remedial action of changing the reader and nanoDot adapter this limit may have been exceeded due to the failing mechanical action of the worn adapter.

**Fig. 6:**
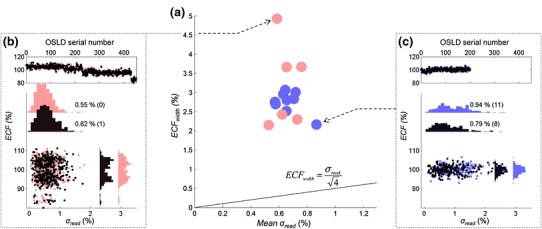
**a** The width of the *ECF* distribution is plotted against mean *σ*_*read*_, where marker size is equal to the average uncertainty in mean *σ*_*read*_, taking into account the extra uncertainty in mean *σ*_*read*_ when two readout sessions are averaged and there is a possible shift in reader state between sessions. The width of the *ECF* distribution varies between 2 and 5 % yet it is not well correlated with mean *σ*_*read*_ of the batch. In the extreme cases, shown in the insets, a readout session may produce, **b** a very wide distribution of *EFC* values with relatively good readout stability, that is, a narrow range of *σ*_*read*_ values, or **c** a narrow *ECF* distribution with poor readout stability. At the *top* of each inset the *ECF* values are plotted sequentially with respect to the OSLDs serial number. OSLDs were chosen and measured randomly therefore serial number has an effect on the *ECF*

If *σ*_*read*_ was the only contributor to the distribution of *ECF* values, that is, if all *ECF*s were equal to unity, and measured *ECF*s were only due to measurement uncertainty, then *ECF*_*width*_ would on average be approximately equal to *σ*_*read*_ divided by the square root of the number of reads. This relationship is plotted as a line in Fig. 6 and the fact that the experimental data lies away from this relationship indicates that the *ECF* variation in a group of dosimeters is greater than the measurement uncertainty. This data strongly re-asserts the advantage of using individual ECFs [6]. Additionally, there is variability in the OSLD stock from one batch to another meaning that one cannot rely on the batches always having a similar range and distribution of ECFs and the width of the ECF peak is not a useful metric to describe the quality of the readout session.

## Conclusion

The continued use of the Landauer Microstar OSLD reader has been analysed over a three and a half year period. This analysis shows that we have observed minor decreases in the quality of the OSLD readouts with small increases in the mean *σ*_*read*_ value. We can attribute these shifts to real variability in the process chiefly the wear on the nanoDot adapter used in the readout of OSLDs. We find that the stability of the reader over this period has been acceptable as there has been no necessity to change the audit outcome scores over this timeframe. The ACDS will continue to monitor the stability of the two readers to prevent an increase in *U*_*read*_ impacting the results of the level one audit. Additionally we observed that the width of the *ECF* distributions can vary by up to 3 %. This variation was not attributed to readout uncertainty but due to an actual variation in the OSLD nanodot stock used by the ACDS.
